# Seasonal Clock Changes Are Underappreciated Health Risks—Also in IBD?

**DOI:** 10.3389/fmed.2019.00103

**Published:** 2019-05-09

**Authors:** Bandik Föh, Torsten Schröder, Henrik Oster, Stefanie Derer, Christian Sina

**Affiliations:** ^1^Institute of Nutritional Medicine, University Hospital Schleswig-Holstein, Lübeck, Germany; ^2^Institute of Neurobiology, University of Lübeck, Lübeck, Germany

**Keywords:** daylight saving time, seasonal clock changes, circadian rhythm disruption, inflammatory bowel diseases, ulcerative colitis, Crohn's disease, European Union

## Abstract

Today, daylight saving time is observed in nearly 80 countries around the world, including the European Union, the USA, Canada, and Russia. The benefits of daylight saving time in energy management have been questioned since it was first introduced during World War I and the latest research has led to varying results. Meanwhile, adverse effects of seasonal time shifts on human biology have been postulated and the European Union is planning to abandon the biannual clock change completely. Medical studies have revealed a correlation of seasonal time shifts with increased incidences of several diseases including stroke, myocardial infarction, and unipolar depressive episodes. Moreover, studies in mice have provided convincing evidence, that circadian rhythm disruption may be involved in the pathogenesis of inflammatory bowel diseases, mainly by disturbing the intestinal barrier integrity. Here, we present previously unpublished data from a large German cohort indicating a correlation of seasonal clock changes and medical leaves due to ulcerative colitis and Crohn's disease. Furthermore, we discuss the health risks of clock changes and the current attempts on reforming daylight saving time from a medical perspective.

## Political Discourse on Daylight Saving Time

Biannual clock changes due to summer time/daylight saving time (DST) are common procedures in most European and North American countries, as well as in parts of South America, Australia, and in New Zealand (1). Historically, George Vernon Hudson, a British/New Zealand entomologist and astronomer, was supposedly the first scientist publicly proposing advantages of a seasonal clock change in 1898 (2). In 1784 however, Benjamin Franklin already discussed a waste of candles due to an extended nightlife in Paris in a curious letter to the *Journal du Paris* suggesting that Parisians need to rise earlier. Although characterized by satiric elements his letter seems to follow the serious purpose of saving candles and decreasing energy expenditure (3). Mainly in an attempt to save resources during World War I, the German Empire and Austria-Hungary were the first countries to introduce DST on 30 April of 1916. Until the end of the war most European countries, Russia, and the US joined them in an unusual case of mutual agreement (4).

In recent years the proposed benefits of DST are being critically discussed. Scientific approaches to evaluate the advantages or disadvantages of DST in terms of energy management have come to inconsistent results. Indeed, there are studies showing minor energy savings due to reduced use of electrical lighting (5–7), whereas others find increased electricity demands for cooling and heating to surpass these savings (8, 9). A 2017 meta-analysis of 44 studies comprehensively states that DST saves ~0.34% of energy in respective countries. As could be expected, there are differences depending on the latitudes of the countries and a larger distance to the equator leads to a higher efficacy of DST (10). Regarding energy management, it may therefore be reasonable to keep seasonal clock changes in countries with high variation of daylight hours, but to abandon them in other regions.

The European Union (EU) has recently conducted a poll asking their citizens whether to maintain or abandon biannual clock changes (11). Reportedly, 4.6 million participants gave their vote setting a record high for public consultations by the EU (12). It is no surprise, that there is public interest in this subject, because seasonal time shifts affect every inhabitant of concerned countries and force them to adjust their biorhythm twice a year. A study from 2015 attempted to measure the welfare effects of time shifts in Germany and Great Britain and reported reduced life satisfaction after the shift to summer time (13). On 31 August of 2018 the European Commission issued a press release stating that 76% of respondents considered the time shifts due to DST a negative or very negative experience corresponding to the aforementioned study. Moreover, 84% of respondents voted for abolishing biannual clock change (14). Although no other public consultation by the European Union generated as many responses, it may still be biased by an overall low participation rate ranging from 0.02% of the population in the United Kingdom (likely reduced by the upcoming Brexit) to 3.79% in Germany. Nevertheless, the European Commission proposed the abandonment of biannual clock changes in favor of permanent summer time to the European Parliament and the Council (14). Very recently, the European Parliament has voted for discontinuing seasonal clock changes. However, before this decision will be executed, negotiations with the responsible EU ministers represented in the Council of the European Union need to be conducted (15).

## Health Risks of Biannual Clock Changes

Circadian rhythm disruption (CRD), as frequently present in our modern 24-h societies, has been suggested to contribute to various diseases. Among them are metabolic (16, 17), cardiovascular (18) and neuropsychiatric disorders (19), as well as different types of cancer (16, 20, 21). More surprisingly, clock changes due to DST are associated with exacerbation of some medical conditions, even though the time shift is only 1 h. Incidences of myocardial infarction were significantly increased on the first 3 days after clock change to summer time in a large Swedish cohort taking into account a time span of 15 years (22). Importantly, this result was later reproduced in five independent studies from Scandinavia, Croatia, Germany, and the USA (23) with a maximum increase of acute infarctions of 24% on Mondays following time shift in spring (24).

Circadian variation in the onset of strokes has been established since the early 1990s by several studies showing increased incidences in the morning (25). More recently, a Finnish study showed elevated hospitalizations due to ischemic stroke in the first 2 days after DST transitions from 2004 to 2013 further supporting time transition-related effects on cardiovascular diseases (26).

Finally, data from the Danish Psychiatric Central Registry show that the transition from summer time to standard time is associated with increased rates of unipolar depressive episodes in the course of 10 weeks (27), implicating that negative effects—at least for depression—might be present for a longer period of time.

As a side note, there is also an ongoing controversy about a possible impact of seasonal time shifts on the rate of traffic accidents. In 1996 *Stanley Coren* published a Correspondence article in the *New England Journal of Medicine* reporting increased numbers of traffic accidents on Mondays following the spring time shift in Canada (28). *Coren's* results are supported by a 2001 study, which reported slightly but significantly increased fatal accidents in the USA following spring time shifts in a data set of 21 years (29). *Coren* speculated that the loss of 1 h of sleep was the underlying cause, because the autumn time shift conversely showed reduced traffic incidents. However, numerous other studies do not support *Coren's* data. Overall, these studies even suggest the seasonal time shift to reduce traffic incidents in the long run, possibly by providing an extra hour of daylight in busier evening traffic hours (30–34).

## Circadian Rhythms and the Gastrointestinal Tract

The two main mechanisms by which circadian regulation of the intestine takes place are vagal afferents from the suprachiasmatic nucleus and intestinal expression of clock genes (35–37). These include, among others, *CLOCK* (Circadian Locomotor Output Cycles Kaput), *BMAL1* (= ARNTL, Aryl hydrocarbon receptor nuclear translocator-like protein 1), *CRY1/2* (cryptochrome 1/2), *PER1-3* (period circadian protein homolog 1–3) and *DBP* (D-site binding protein), which are key factors in self-sustaining transcriptional-translational feedback loops (TTFL) that constitute the foundation of circadian regulation on a molecular level within mammalian cells (38–41). In brief, CLOCK and BMAL1 proteins form a heterodimeric transcription factor inducing the expression of their own negative regulators PER1-3 and CRY1-2 in a negative TTFL. PER and CRY proteins accumulate in the cytoplasm and are phosphorylated by casein kinase Iε (CKIε) and glycogen synthase kinase-3 (GSK3). Subsequently, they translocate back to the nucleus, where they repress transcriptional activation by their own activators CLOCK and BMAL1. Gradually, PER and CRY proteins are degraded closing the loop and allowing the cycle to start anew (42). An accessory feedback loop depends on REV-ERBα/ß (= NR1D1/2, nuclear receptor subfamily 1, group D, member 1/2) and RORα/ß (= NR1F1/2, nuclear receptor subfamily 1, group F, member 1/2), which are expressed in a circadian rhythm in intestinal tissue as well, regulating transcription of *BMAL1* (38).

These circadian clock gene/protein oscillations need to be relayed to the expression of effector genes in order to exert functional effects in peripheral organs. DBP is considered a prototypic local transducer of these signals, which was shown in liver tissue, where it affects the circadian expression of cytochrome P450 enzymes (43, 44). In recent years, it has become evident that post-translational modification of the aforementioned gene products is an additional key factor in regulating cellular clock rhythms (42). In the intestinal tract, several clock genes are preferentially expressed in epithelial cells and in the enteric nervous system implying a relevant role in the coordination of intestinal functions (39, 45). Microarray analysis show that ~4% of all distal colonic genes are expressed with circadian rhythms many of which are involved in cell signaling, proliferation, inflammation, intestinal motility, and secretion (46). Indeed, gastrointestinal motility (45), gastric acid secretion (47), intestinal regeneration (48), activity of mucosal enzymes and carbohydrate as well as peptide absorption have been shown to be regulated in a circadian manner (37, 49–52).

Additionally, the host's circadian rhythm regulates diurnal variations of gut microbiota (53). Microbial cues, on the other hand, are transformed into rhythmic downstream signals by oscillating expression of Toll-like receptors in intestinal epithelial cells. These signals result in a tightly regulated circadian expression profile of various genes with crucial functions for metabolic and immunologic homeostasis in the intestine (41, 54), representing a novel facet in bidirectional communication of host and gut microbiota. Disruption of the host's circadian rhythm by varying mechanisms can lead to intestinal dysbiosis, symptoms of irritable bowel syndrome, metabolic dysregulation, increased glucose tolerance, and obesity among others (45, 53–58).

Notably, circadian rhythm disruption (CRD) also induces increased intestinal permeability—a major culprit in metabolic liver and inflammatory bowel diseases (IBD) (59). An early study by Preuss et al. providing limited mechanistic insight showed that shifting the light-dark cycle exacerbates experimental colitis dramatically (60). Accordingly, genetic ablation of one of the key factors of the accessory TTFL, REV-ERBα, leads to increased susceptibility to experimental colitis in mice, whereas mice with increased REV-ERBα activation are protected (61). Moreover, we have recently shown that both genetically (Per1/Per2-mutant mice) and externally induced CRD exacerbates mucosal inflammation via inhibition of intestinal epithelial cell proliferation and induction of necroptosis in a well-established murine model of IBD (62). By demonstrating that sleep deprivation, which is often linked to CRD, worsens colonic inflammation, additional evidence for an important role of the circadian rhythm in intestinal homeostasis was provided (63).

In human IBD circadian genes are downregulated in inflamed and non-inflamed mucosal biopsies and systemically in mononuclear blood cells, correlating with increased markers of inflammation and disease activity (64). Another study from 2015 shows a deregulation of key circadian genes in the mucosa of IBD patients including *CRY1, PER1*, and *PER3* using genome-wide cDNA microarray analysis (65). Accordingly, CRD is suggested as a possible environmental trigger of IBD activation (66, 67). This is intriguing, considering that various environmental factors including psychosomatic disorders, antibiotics, gastrointestinal and upper respiratory infections and stress have been proposed as major triggers of relapse, but lack consistent correlations in prospective studies (68–70). To date, the most reliable clinical predictors for acute flares remain high relapse rates in the past, a short time since the last relapse and disease severity in general, providing only limited mechanistic insight (68, 71).

**Figure 1:**
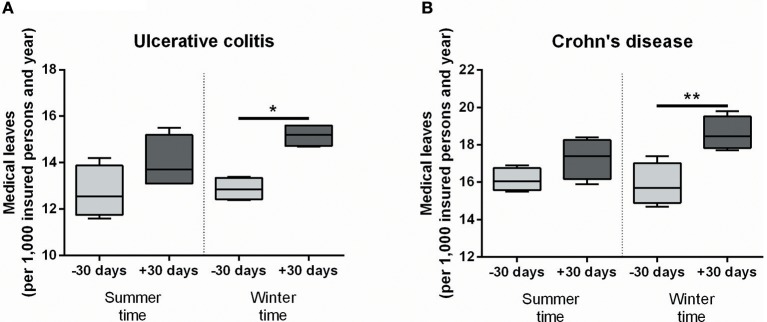
Medical leave frequencies due to IBD increase after seasonal clock changes. Medical leaves are shown per 1,000 insurance holders in the 30 days before and after seasonal clock changes for ulcerative colitis **(A)** and Crohn's disease **(B)** as defined by the International Classification of Diseases (ICD-10). Statistical testing was performed using One-Way ANOVA followed by Tukey's honest significant difference test in Prism 6 (GraphPad Software). *P < 0.05; **P < 0.01.

We analyzed medical leave frequencies due to ulcerative colitis (UC) and Crohn's disease (CD) 30 days before and after seasonal time shifts throughout the years 2010 to 2013 in a large cohort of more than 10 million insurance holders of a German health insurance company. Strikingly, after the autumn time shift significantly more UC patients reported sick leaves (Figure 1A, 12.88/1,000 insurance holders vs. 15.18/1,000, *P* = 0.0135). Similarly, medical leave frequency for CD patients was significantly increased in the same period of time (Figure 1B, 15.88/1,000 vs. 18.6/1,000, *P* = 0.0077). The spring time shift led to increased medical leave rates for both disease entities as well, but the differences were not statistically significant (Figures 1A,B). Although the absolute changes are modest and medical leave frequencies are only a surrogate parameter for acute IBD flares, these results indicate a relevant effect of seasonal clock change on symptom severity of IBD patients forcing them to take a medical leave.

Previously published data on increases of IBD flares in the autumn/winter months compared to spring are partially reproduced by our data, although the surrogate parameters differ from study to study (68, 71, 72). Climatic differences as well as sunlight exposure and associated vitamin D activation are possible causes for these seasonal variations and may partially explain the here observed increase after the autumn time shift, but certainly not after the clock change in spring, when sunlight exposition increases and temperatures rise (73, 74). Notably, the variations in relapse frequency observed in these studies are predominantly registered months before the dates of clock changes and do not coincide with them (68, 72). It is therefore unlikely that increased medical leave rates after seasonal clock changes in our data are merely caused by the change of seasons. Furthermore, it should be noted that several other studies did not report seasonality or even conversely registered a rise of IBD flares in spring (75–77). Possible causes for the varying results include the definition of flares, different geographical and genetic backgrounds of study populations and environmental factors such as infections, climate, and food (78).

Many intestinal diseases among them IBD may be heavily influenced by psychological factors. Therefore, it is important to consider negative psychosomatic effects of seasonal time shifts as effectors in our data, as reflected by increased depressive episodes after autumn time shift (27). Nevertheless, our data are coherent with abundant experimental evidence of a crucial role of circadian rhythm in the maintenance of intestinal homeostasis and barrier function. Although we are not able to distinguish between immunologic and psychosomatic mechanisms underlying our results, these data may ultimately imply socioeconomic costs of biannual clock changes as increased medical leaves entail fewer working hours. Future prospective studies measuring hospitalization rates and disease activity scores of IBD patients are, however, needed to conclusively support an effect of seasonal clock changes on IBD relapse rates.

## Conclusion

DST is a highly polarizing issue, concerning most people in western civilizations. Data on energy savings due to DST are inconsistent, but DST appears more favorable in countries located remote from the equator. Epidemiological data suggest a link of seasonal time changes to increased incidences of acute myocardial infarction, ischemic stroke, and depression. We here provide additional data indicating a correlation of seasonal clock changes with a surrogate parameter of acute IBD flares, corroborated by existing experimental evidence on circadian regulation of the intestinal homeostasis in mice and humans. Further prospective studies will be needed to further support these results. From a medical perspective—and setting aside small energy savings and highly questionable effects on traffic incidents—the abolishment of biannual clock changes should be seriously considered. Following a public consultation, the European Union is currently evaluating a permanent switch to summer time and it would not be surprising if other regions were following this example in the years to come.

## Author Contributions

BF analyzed and interpreted the data and drafted the manuscript. TS was a major contributor in writing the manuscript. HO provided important intellectual content. SD analyzed and interpreted the data. CS was a major contributor in writing the script and provided important intellectual content. All authors read and approved the final manuscript.

### Conflict of Interest Statement

The authors declare that the research was conducted in the absence of any commercial or financial relationships that could be construed as a potential conflict of interest.

## Abbreviations

ARNTL/BMAL1: Aryl hydrocarbon receptor nuclear translocator-like protein 1
CKIε: casein kinase Iε
CLOCK: Circadian Locomotor Output Cycles Kaput
CD: Crohn's disease
CRD: circadian rhythm disruption
CRY1: cryptochrome 1
DBP: D-site binding protein
DST: daylight saving time/summer time
GSK3: glycogen synthase kinase-3
IBD: inflammatory bowel diseases
NR1D1/2 (REV-ERBα/β), nuclear receptor subfamily 1, group D: member 1/2
NR1F1/2 (RORα), nuclear receptor subfamily 1, group F: member 1/2 (RAR-related orphan receptor alpha/beta)
PER1-3: period circadian protein homolog 1
TTFL: transcriptional-translational feedback loop
UC: ulcerative colitis.
